# Phenoscape: Identifying Candidate Genes for Evolutionary Phenotypes

**DOI:** 10.1093/molbev/msv223

**Published:** 2015-10-24

**Authors:** Richard C. Edmunds, Baofeng Su, James P. Balhoff, B. Frank Eames, Wasila M. Dahdul, Hilmar Lapp, John G. Lundberg, Todd J. Vision, Rex A. Dunham, Paula M. Mabee, Monte Westerfield

**Affiliations:** ^1^Institute of Neuroscience, University of Oregon; ^2^School of Fisheries, Aquaculture and Aquatic Sciences, Auburn University; ^3^National Evolutionary Synthesis Center, Durham, NC; ^4^Department of Anatomy and Cell Biology, University of Saskatchewan, Saskatoon, SK, Canada; ^5^Department of Biology, University of South Dakota; ^6^Department of Ichthyology, The Academy of Natural Sciences, Philadelphia, Philadelphia, PA; ^7^Department of Biology, University of North Carolina, Chapel Hill

**Keywords:** molecular evolution, gene expression, evolutionary phenotypes, catfish, nonmodel organism

## Abstract

Phenotypes resulting from mutations in genetic model organisms can help reveal candidate genes for evolutionarily important phenotypic changes in related taxa. Although testing candidate gene hypotheses experimentally in nonmodel organisms is typically difficult, ontology-driven information systems can help generate testable hypotheses about developmental processes in experimentally tractable organisms. Here, we tested candidate gene hypotheses suggested by expert use of the Phenoscape Knowledgebase, specifically looking for genes that are candidates responsible for evolutionarily interesting phenotypes in the ostariophysan fishes that bear resemblance to mutant phenotypes in zebrafish. For this, we searched ZFIN for genetic perturbations that result in either loss of basihyal element or loss of scales phenotypes, because these are the ancestral phenotypes observed in catfishes (Siluriformes). We tested the identified candidate genes by examining their endogenous expression patterns in the channel catfish, *Ictalurus punctatus*. The experimental results were consistent with the hypotheses that these features evolved through disruption in developmental pathways at, or upstream of, *brpf1* and *eda*/*edar* for the ancestral losses of basihyal element and scales, respectively. These results demonstrate that ontological annotations of the phenotypic effects of genetic alterations in model organisms, when aggregated within a knowledgebase, can be used effectively to generate testable, and useful, hypotheses about evolutionary changes in morphology.

## Introduction

Identifying the genetic and developmental changes that underlie the morphological diversification of life is an ultimate goal of many research programs in developmental and evolutionary biology. Some of the most spectacular advances in biology in the past decade have been gained through this approach, including the discovery that genes, genetic architectures, expression patterns, networks, and developmental processes are highly conserved, well beyond expectation, even across very distantly related organisms (Degnan 2010).

Although our knowledge has deepened about the bases of phenotypic divergence along major aspects of organismal architectures (e.g., flower patterning; body axes and segment patterning), the developmental genetic basis for most of the known phenotypic transitions in the evolution of life remains unexplored nonetheless. One reason for this is that laboratories often focus on a single gene or gene network in one (or few) model taxa to pinpoint candidate genes that are known from other (model) species. Ideally, however, an investigator could take the set of novel features for a clade and query model organism databases for similar phenotypes resulting from genetic perturbations. The roles of these genes in the evolutionary phenotype could then be tested experimentally, provided an experimentally tractable system exists.

A data-driven approach for generating candidate gene hypotheses requires the capacity to process computationally the vast number of model organism phenotypes and large ontologies that allow software to recognize similarities and relationships among phenotypes regardless of what vocabulary researchers use to describe them (Mabee, Ashburner, et al. 2007). In recent years, inspired by the success of the Gene Ontology (GO) in enabling computation over descriptive information about gene function (Blake and Harris 2008), ontologies have been developed and fruitfully applied to phenotypic data (Deans et al. 2015). Though developed initially for use in disease gene discovery (Washington et al. 2009; Hoehndorf et al. 2011; Oellrich et al. 2012), ontologies such as Uberon (Haendel et al. 2014) and Phenotype and Trait Ontology (PATO) (Gkoutos, Green, Mallon, Blake, et al. 2004) empower researchers to imagine a much wider variety of computational applications for descriptive phenotype information.

Here, we demonstrate that compilations of data describing the phenotypic effects of genetic alterations in model organisms can be leveraged to generate hypotheses about evolutionary changes in morphology. To do this, we took advantage of the Phenoscape Knowledgebase (http://kb.phenoscape.org), an ontology-driven data resource containing information about both mutant zebrafish (*Danio rerio*) phenotypes curated by the zebrafish model organism database (ZFIN, http://zfin.org; Ruzicka et al. 2015) and evolutionary phenotype variation in ostariophysan fishes, the group of fishes that includes zebrafish, as documented in the comparative morphology and systematics literature (Mabee, Arratia, et al. 2007). We used the Phenoscape Knowledgebase to generate hypotheses about candidate genes for experimentally tractable evolutionary phenotypes (see Materials and Methods), which yielded candidate genes for two striking phenotypes that are characteristic of the diverse, ecologically and economically important clade of catfishes (Siluriformes) with over 3,600 living species: 1) Loss of the “tongue” or basihyal element (i.e., cartilage and bone; hereafter referred to as basihyal; Arratia and Schultze 1990; de Pinna 1993) and 2) loss of scales (Fink SV and Fink WL 1981). Here we present, for the first time, endogenous expression patterns of candidate genes potentially involved with the evolutionary loss of basihyal (bromodomain and PHD finger containing 1*, brpf1*; Laue et al. 2008) and scales (ectodysplasin A, *eda*; ectodysplasin A receptor, *edar*; Harris et al. 2008) using whole mount and cryosection in situ hybridization, respectively.

**Fig. 1.**
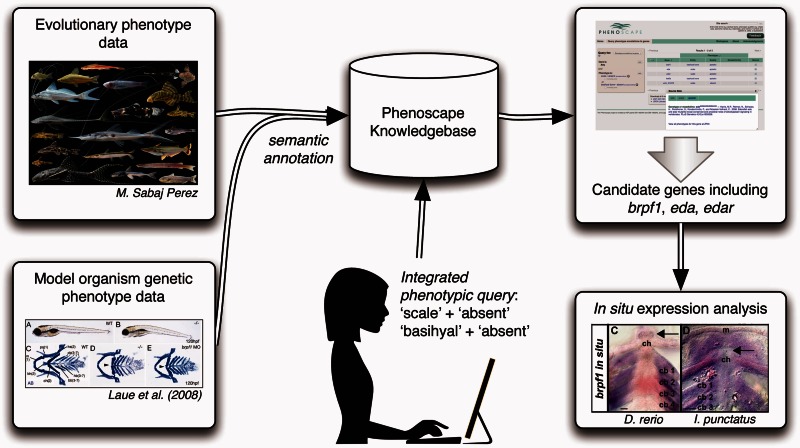
Flow chart showing computational and experimental steps used to propose and test candidate genes for evolutionary phenotypic novelties. Evolutionary phenotype data for fish species and model organism genetic phenotype data for zebrafish (from ZFIN) are semantically annotated and housed in the Phenoscape Knowledgebase. A user query to the Knowledgebase for genes associated with evolutionary phenotypes of interest (here, scales absent and basihyal absent) returns a list of candidate genes based on the model organism data. These in silico candidates can be experimentally assessed (e.g., in situ gene expression analysis using *Danio rerio* and *Ictalurus punctatus*).

We tested the ability of the Phenoscape Knowledgebase to identify candidate genes for evolutionary phenotypes in experimentally accessible organisms (fig. 1). For this, we used the commercially important channel catfish (*Ictalurus punctatus*) as a tractable representative of Siluriformes. Although no catfish species are routinely used for developmental studies, *I. punctatus* is widespread throughout North America, commercially bred in aquaculture (USDA 2005), and has EST (expressed sequence tag) sequences (Li et al. 2007) as well as database resources (cBARBEL; Lu et al. 2011) available*.* Moreover, *I. punctatus* has been used previously for molecular (Waldbieser et al. 2001; Li and Waldbieser 2006; Xu et al. 2006) and immune system investigations (Kocabas et al. 2002; Bao et al. 2006; Baoprasertkul et al. 2006; Peatman et al. 2007). Importantly, *I. punctatus* can be considered a viable candidate for in situ gene expression studies (Steinke et al. 2006; Chen et al. 2010; Wang et al. 2010; Liu 2011; Liu et al. 2011; Ninwichian et al. 2012; Jiang et al. 2013; Zhang et al. 2013).

## Results

### In Silico Analysis Identifies Candidate Genes for Evolutionary Phenotypes

As is the case for most nonmodel taxa, necessary baseline information about developmental staging and morphology (Gilbert 2009; Rowan et al. 2011), specifically the timing of pharyngeal arch chondrification, was unavailable. In light of this, samples were collected across developmental time-points for the channel catfish (*I. punctatus*), South American cave-dwelling suckermouth armored catfish (*A.* cf. *triradiatus*), and armored bronze corydoras catfish (*Corydoras aeneus*) to establish the species-specific developmental timing of pharyngeal arch chondrification. Both *A.* cf. *triradiatus* and *C. aeneus* were readily available and have been used previously in studies of jaw development (Geerinckx et al. 2005, 2007; Geerinckx and Adriaens 2007, 2008) and jaw morphology (Huysentruyt et al. 2007, 2008, 2009, 2011), respectively.

**Fig. 2.**
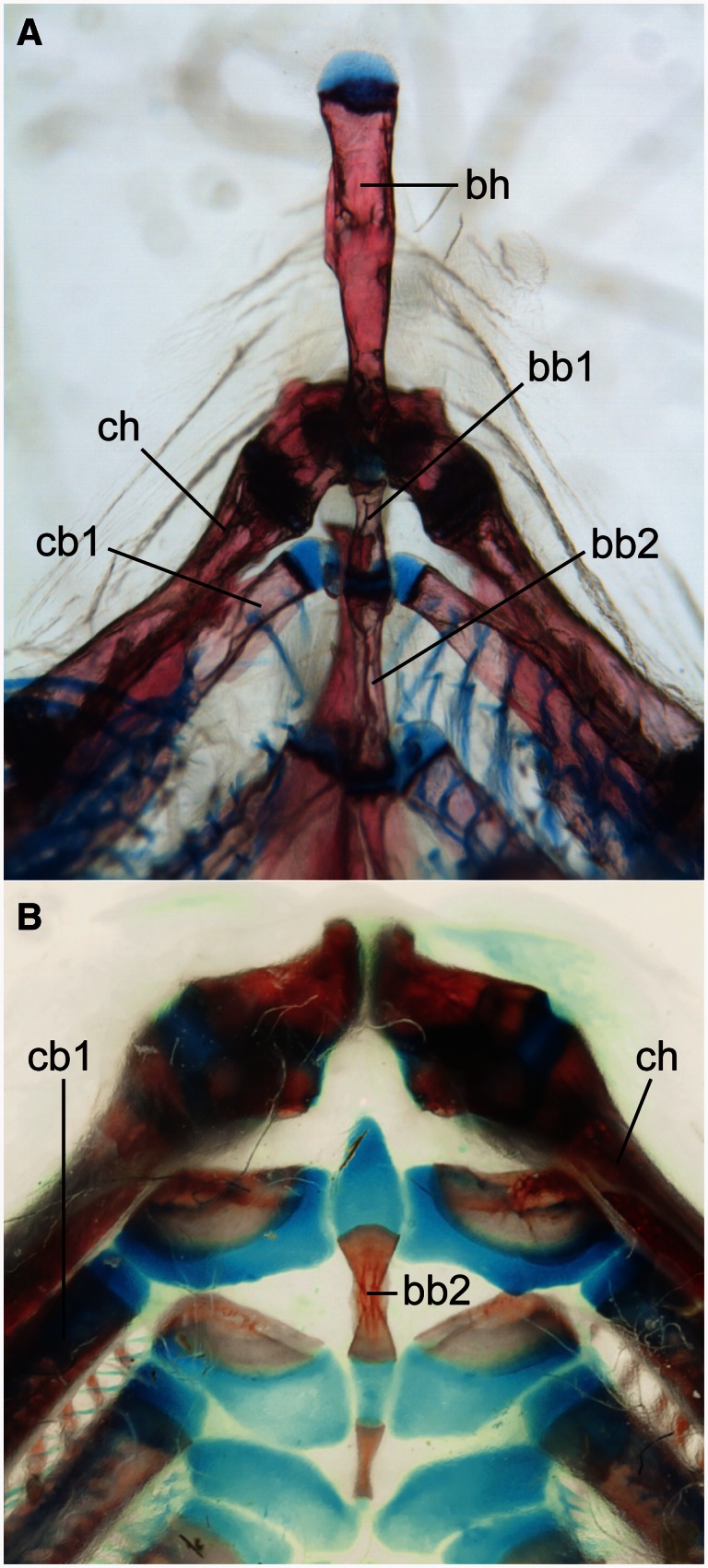
Loss of basihyal element in catfishes. Catfishes (Siluriformes) are characterized by the loss of the basihyal element in contrast to relatives, including zebrafish. (*A*) Zebrafish, *Danio rerio* (purchased from an aquarium fish store by P. Mabee). (*B*) Spotted Bullhead Catfish, *Ameiurus serracanthus* (ANSP 185358; provided by K. Luckenbill). Images show lower branchial elements in ventral view. bb, basibranchial; bh, basihyal; cb, ceratobranchial; ch, ceratohyal. Scale bars = 100 µm.

**Fig. 3.**
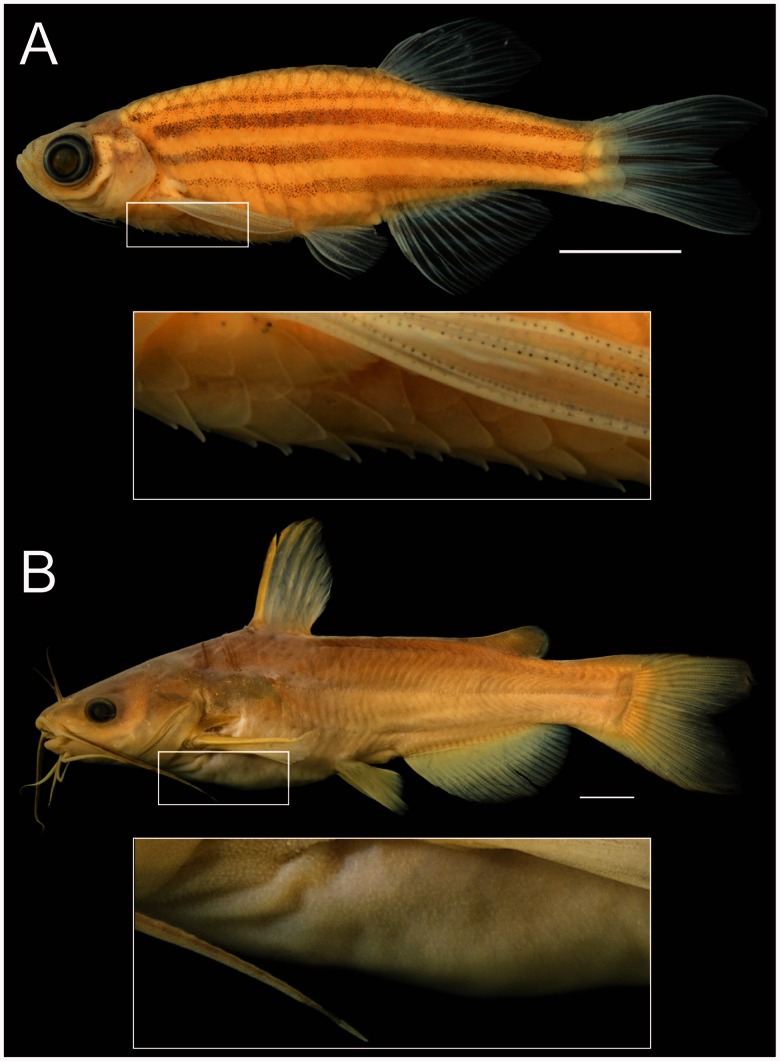
Loss of scales in catfishes. Catfishes (Siluriformes) are characterized by the loss of the scales in contrast to relatives, including zebrafish. (*A*) Zebrafish, *Danio rerio* (ANSP 189304; provided by K. Luckenbill) in lateral view with close up of scales. (*B*) White Catfish, *Ameiurus catus* (ANSP 11678; provided by M. A. Arce-Hernandez) in lateral view with close up of skin. Note that the bumps on the skin visible in the insert are a variety of soft-tissue structures all without bone, likely including externalized taste buds, free-neuromasts, and integumentary glands. Scale bars = 1 cm.

Two prominent phenotypic changes that distinguish catfishes from other ostariophysan fishes are the absence of a basihyal (“tongue”; fig. 2) and the absence of elasmoid scales that characterize most actinopterygian fishes (fig. 3; Fink SV and Fink WL 1981; Arratia and Schultze 1990; de Pinna 1998; Sire and Akimenko 2004). The scutes (i.e., postcranial dermal plates of armored catfishes, e.g., Callichthyidae, Loricariidae, Doradidae, etc.; Sire and Huysseune 2003) develop differently from elasmoid scales (Sire 1993) and are a derived condition within catfishes (Fink SV and Fink WL 1996). Using the Phenoscape Knowledgebase, we sought genes that could be candidates responsible for these phenotypic differences in the zebrafish model based on genetic and phenotype data from ZFIN. Gene phenotype–taxon phenotype associations were generated from the Phenoscape Knowledgebase using matching anatomical entities, and then examined by hand. The gene phenotype annotations we obtained from ZFIN associate an aplastic or absent basihyal phenotype with the disruption of 11 zebrafish genes, including bromodomain and PHD finger containing 1 (*brpf1*; Laue et al. 2008), disrupted in schizophrenia 1 (*disc1;* Wood et al. 2009), dispatched homolog 1 (*disp1*; Schwend and Ahlgren 2009), facelift (*fac*; Schilling et al. 1996), forkhead box D3 (*foxd3*; Neuhauss et al. 1996), heart and neural crest derivatives expressed 2 (*hand2*; Miller et al. 2003), K(lysine) acetyltransferase 6A (*kat6a*; Laue et al. 2008), SRY (sex determining region Y)-box 9a (*sox9a*; Yan et al. 2002), unnamed th9 (*unm_th9*), unnamed tn20c (*unm_tn20c*), and unnamed ty5 (*unm_ty5*). We selected *brpf1* as a candidate gene because mutation or knockdown in *D. rerio* results in phenotypes similar to known features of catfishes (e.g., loss of basihyal; Laue et al. 2008). An aplastic or absent scale is associated with the disruption of three zebrafish genes, including *eda* and *edar* (Harris et al. 2008), and unnamed t31273 (*unm_t31273*), which is a gene of unknown function; therefore, we selected *eda* and *edar* as candidates for investigating scale loss in catfish because they are experimentally tractable given their known functions in zebrafish.

### Developmental Morphology and Gene Expression Indicate the Roles of Candidate Genes in Evolutionary Phenotypes

**Fig. 4.**
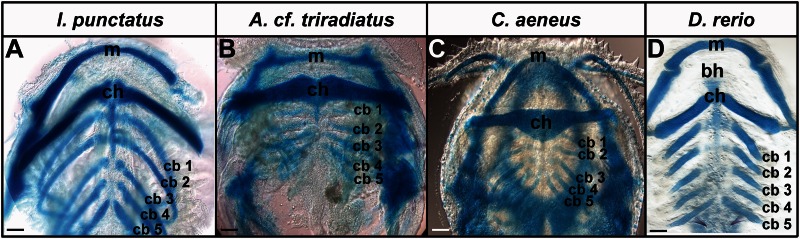
Pharyngeal arches develop later in catfish than in zebrafish. Chondrification of jaw (mandibular arch), hyoid arch, and branchial arches as detected by Alcian Blue staining. Images show lower branchial elements in ventral view. (*A*) *Ictalurus punctatus*, 96 hpf; (*B*) *Ancistrus* cf. *triradiatus*, 96 hpf; (*C*) *Corydoras aeneus,* 102 hpf; (*D*) *Danio rerio*, 72 hpf. m, Meckel’s cartilage; bh, basihyal; ch, ceratohyal cartilage; cb, ceratobranchial cartilages. Scale bars = 100 µm.

Alcian Blue staining of embryonic *I. punctatus, A.* cf. *triradiatus,* and *C. aeneus* revealed complete chondrification of all arches (mandibular, hyoid, and ceratobranchials 1–5) by 96, 96, and 102 h postfertilization (hpf), respectively (fig. 4).

Because *eda, edar,* and *brpf1* expression had not previously been investigated in catfishes, we verified the presence of transcripts in all three species by reverse transcription polymerase chain reaction (RT-PCR) across early developmental stages (24–120 hpf; data not shown). Sequencing and ClustalW alignment (Larkin et al. 2007) of RT-PCR products confirmed higher levels of *eda* and *edar* nucleotide sequence similarity (i.e., identical nucleotides) among catfish species (97–100% similar) than between catfishes and zebrafish (74–76% similar). Lower levels of nucleotide sequence similarity were observed for *brpf1* sequences among catfishes (71–86% similar) than between catfishes and zebrafish (72–73% similar).

**Fig. 5.**
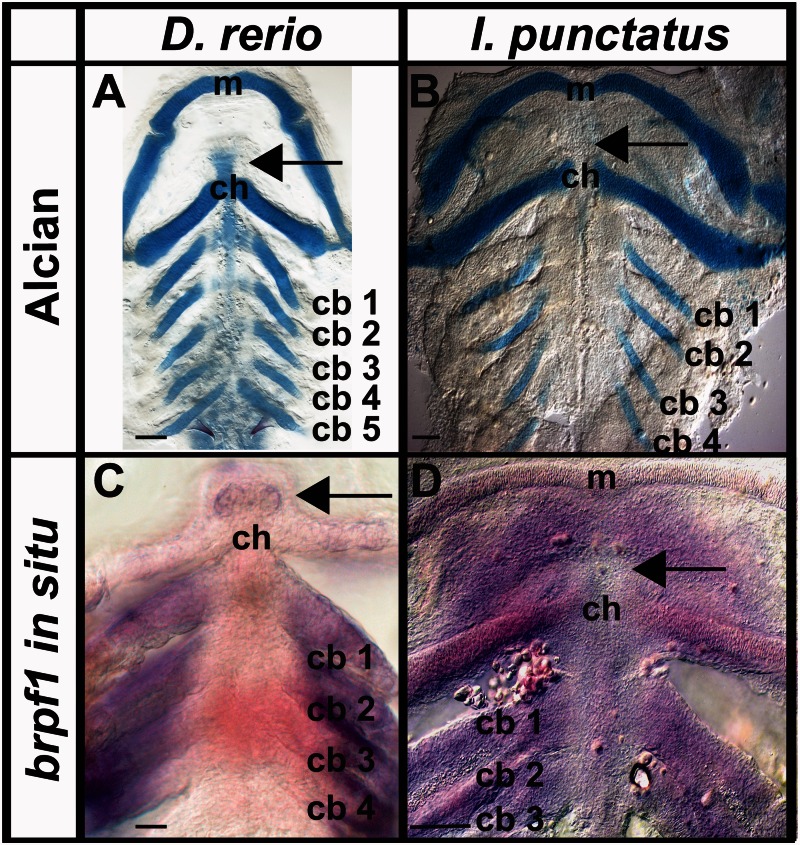
Catfishes lack *brpf1* expression in the region where the basihyal forms in zebrafish. Expression of *brpf1* during pharyngeal arch skeletal development in *Danio rerio* and *Ictalurus punctatus*, as detected by whole-mount in situ hybridization. Staining of pharyngeal arch cartilages using (*A*) Alcian Blue in *D. rerio* (72 hpf) and (*B*) Alcian Green in *I. punctatus* (86 hpf). Endogenous *brpf1* expression in (*C*) *D. rerio* (72 hpf) and (*D*) *I. punctatus* (86 hpf). Arrows indicate location of basihyal element in *D. rerio* (cypriniform) and lack of basihyal element in *I. punctatus* (siluriform). Images are representative of ≥30 embryos. No labeling was detected in negative controls (data not shown). m, Meckel’s cartilage; ch, ceratohyal cartilage; cb, ceratobranchial cartilages. Scale bars = 100 µm.

We used Alcian Blue and Alcian Green staining to establish 72 and 86 hpf as key time-points in *D. rerio* and *I. punctatus* early pharyngeal arch skeletal development (i.e., chondrification of certatobranchials 1–4 and midline cartilages; fig. 5*A* and *B*; Eames et al. 2013), respectively. We then targeted these developmental time-points for investigating the loss of basihyal formation as it relates to endogenous *brpf1* expression in a representative cypriniform (basihyal present) and siluriform (basihyal absent) species (fig. 5*C* and *D*), respectively. *Brpf1* expression was observed in all arches of *D. rerio* by 72 hpf and *I. punctatus* embryos by 86 hpf, as expected (fig. 5*C* and *D*; Laue et al. 2008). However, *brpf1* expression was absent in the midline of the hyoid arch (fig. 5*D*), where the basihyal normally forms in nonsiluriform fishes (e.g., *D. rerio*, fig. 5*C*; Cubbage and Mabee 1996; Parichy et al. 2009; Mabee et al. 2011). To ensure that there were actually cells in this region that failed to express *brpf1* in *I. punctatus*, we counterstained embryos after in situ hybridization with Toluidine Blue and removed the pharyngeal arches by dissection. This method confirmed the presence of cells in the anterior hyoid region at these developmental time-points (data not shown). These experiments establish, for the first time, that *brpf1* expression is absent in cells immediately anterior to the hyoid arch in *I. punctatus* during pharyngeal arch development, whereas the basihyal is present and expresses *brpf1* in nonsiluriform fishes (e.g., *D. rerio*; Laue et al. 2008).

**Fig. 6.**
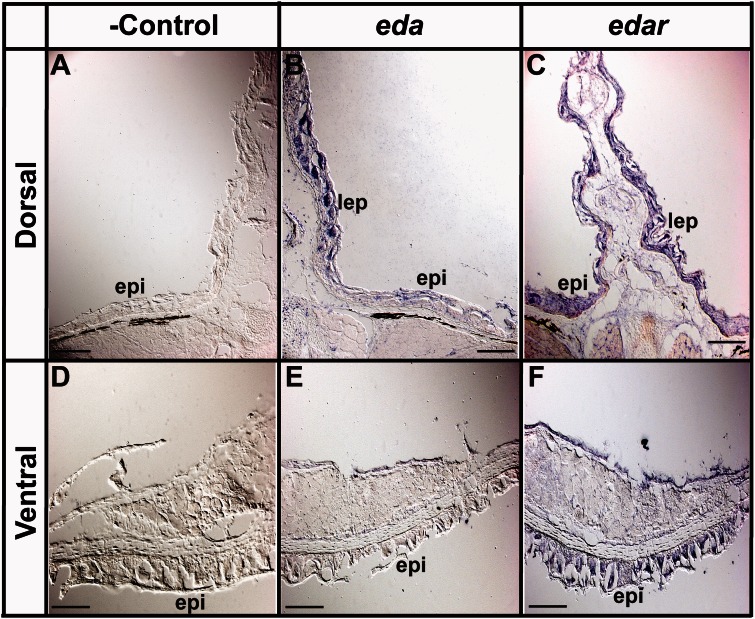
Epidermis of 4-week-old *Ictalurus punctatus* lacks *eda* expression. In situ hybridization using DIG-labeled riboprobes on tissue cryosections was used to determine the patterning of endogenous *eda* and *edar* expression (see Materials and Methods). (*A*, *D*) Negative hybridization control for dorsal and ventral epidermal tissues, respectively. (*B*, *C*) Expression of *eda* and *edar* in dorsal fin lepidotrichia as expected (Harris et al. 2008). (*E*, *F*) Absent and present endogenous expression of *eda* and *edar* in ventral epidermal tissues, respectively. Images are representative of ≥10 cryosectioned juveniles. epi, epidermis; lep, lepidotrichia. Scale bars = 100 µm.

In 4-week-old *I. punctatus* juveniles, both *eda* and *edar* are expressed in dorsal fin lepidotrichia, as expected (fig. 6*B* and *C*; Harris et al. 2008). We also detected robust *edar* expression in the dorsal and ventral epidermis (fig. 6*C* and *F*); however, robust *eda* expression was not detected in the dorsal and ventral epidermis (fig. 6*B* and *E*). Because both *eda* and *edar* are strongly expressed in zebrafish epidermis (Harris et al. 2008), these in situ hybridization experiments demonstrate that *eda* expression has been reduced, or potentially silenced, in epidermal cells of juvenile *I. punctatus*. These results are consistent with the hypothesis that modification of the *eda* signaling pathway (i.e., loss of ligand expression but not receptor expression) underlies the evolutionary loss of scales in Siluriformes.

## Discussion

A central quest in biology is to understand how the diversity of phenotypes is regulated genetically. Although it has long been possible to comb the literature to find individual candidate genes of interest for a phenotype observed in a different taxon, recent efforts to scale up the capture of both genetic and phenotypic variation (Binder and Zon 2013), and the online accessibility of information from these studies (ZFIN; Ruzicka et al. 2015), provide a strong motivation to develop computational tools that support efficient exploration of causal linkages between genes and phenotypes. Genetic and phenotypic comparisons of phylogenetically disparate model organisms have revealed oftentimes surprisingly deep conservation of the role of genes in developmental processes, as well as an expectation that similar phenotypes (homologous or not) are likely to share genetic pathways and possibly common regulators (e.g., Ritter et al. 2010; Gallant et al. 2014). Techniques for testing developmental genetic hypotheses in nonmodel organisms are becoming increasingly sophisticated, providing experimental investigation of candidate genes for a much wider variety of phenotypes in nature (Abzhanov et al. 2008).

The Phenoscape Knowledgebase, which integrates the peer-reviewed fish morphology literature with zebrafish model organism database data (phenotypes and genotypes), allows evo-devo researchers to develop hypotheses that otherwise would be impossible to generate manually because of the immensity of information (Manda et al. 2015). Currently, morphology data are not present in any other database in a form that can be compared directly with model organism data. The Phenoscape Knowledgebase expedites the process of hypothesis generation and expands it to a scale never before possible. Here, we have demonstrated the viability of such an approach for mining existing genotype–phenotype information computationally to generate candidate genes for phenotypes in a nonmodel organism. The candidate genes *brpf1* and *eda*/*edar* were predicted by the Phenoscape Knowledgebase to play roles in the evolutionary losses of the basihyal and scales in Siluriformes, respectively. These computational predictions were empirically tested by examining the expression patterns of candidate genes in situ in an experimentally tractable representative of Siluriformes, the channel catfish (*I. punctatus*).

To investigate the evolutionary losses of the basihyal and scales in Siluriformes, we characterized the timing of pharyngeal arch development in three catfish species (*I. punctatus, C. aeneus*, and *A.* cf. *triradiatus*) as well as the pattern of endogenous *brpf1* expression in embryonic and juvenile *I. punctatus*, and compared these patterns to zebrafish. Although all catfishes lack a basihyal, little else is known about their pharyngeal arch development. Zebrafish develop a basihyal by 72 hpf (Cubbage and Mabee 1996; Parichy et al. 2009; Engeman and Mabee 2012), and our analysis suggested that pharyngeal arch development has reached a similar developmental stage by 78–86 hpf in *I. punctatus.* Silencing *brpf1*, which encodes a MOZ histone acetyl transferase complex subunit, prevents basihyal formation and generates a broader and flattened mouth in loss-of-function zebrafish mutants (Laue et al. 2008). Loss of function of *brpf1* in medaka (*Oryzias latipes*) *bis* mutants results in similar developmental craniofacial skeletal patterning defects, most notably the reduction in basihyal length (Hibiya et al. 2009). We found that *I. punctatus* lacks *brpf1* expression in cells in the location where the basihyal forms in these other species. The absence of *brpf1* expression in developing *I. punctatus* midline cells of the anterior hyoid region and its presence in the pharyngeal arches supports our hypothesis that loss of *brpf1* expression (or an upstream regulator) may be mechanistically responsible for the loss of basihyal formation in the catfish lineage. One possible explanation is that *brpf1* expression may be repressed in the anterior hyoid region by way of *cis*- or *trans*-acting regulators that are evolutionarily unique to Siluriformes. This hypothesis could be tested by examining the endogenous expression of associated *Hox* genes (Hibiya et al. 2009) in the craniofacial tissues of *I. punctatus*.

All catfishes also lack true scales and, instead, have a scaleless epidermis (Grizzle et al. 1976; Sire 1993; Konrádsdóttir et al. 2009; Burns et al. 2010). Zebrafish normally form scales, but mutations in the *eda* or *edar* genes that encode the ectodysplasin A signaling protein and receptor, respectively, result in failure of scale formation (Harris et al. 2008). Loss of scales was also observed in medaka (*O. latipes*) following mutational silencing of the *rs-3* locus, which encodes the *eda* receptor protein EDAR (Kondo et al. 2001). Likewise, *eda* has been associated with variation in scale (postcranial dermal element) number of the three-spine stickleback (*Gasterosteus aculeatus*) that has undergone repeated selection in freshwater populations worldwide (Colosimo et al. 2005; Bell et al. 2010). We found that although cells of the epidermis in *I. punctatus* express *edar*, they fail to express *eda* (fig. 6).

Given the protein sequence homology for both EDA and EDAR among catfishes, which is consistent with previous observations that the EDA signaling pathway is evolutionarily conserved (Colosimo et al. 2005; Harris et al. 2008), we investigated whether an alteration in the *eda* signaling pathway underlies evolutionary scale loss in *I. punctatus*. The *eda* and *edar* expression patterns observed in epidermis adjacent to dorsal fin (weak and strong) and ventral epidermis (undetectable and detectable) support our hypothesis that a change in *eda* signaling pathway balance (i.e., reduction of ligand, but not receptor, expression) underlies this evolutionary difference, respectively. The reduction of *eda* ligand expression would potentially prevent organization of epithelial cells into signaling centers and dermal placodes and, in doing so, prevent basal epidermis fibroblasts from assembling during early stages of scale formation (Kondo et al. 2001; Colosimo et al. 2005; Bell et al. 2010). This putative mechanism would instead generate the scaleless (i.e., naked) epidermis characteristic of Siluriformes. In contrast, we found expression of *edar* in epidermal cells in zebrafish, consistent with a previous study (Harris et al. 2008). Presumably, the signaling through EDAR required for scale development is likely to be functionally inhibited by the reduction of the EDA ligand. Changes in the regulation of *eda* (e.g., transcriptionally or epigenetically) could potentially underlie the undetectable level of expression in juvenile *I. punctatus* epidermis.

These experimental data demonstrate that in silico hypotheses generated through ontology-driven searches of linked gene-phenotype data in model organisms can be leveraged to discover genes involved in evolutionary changes in morphology. Further development of methods (e.g., artificial intelligence; Gil et al. 2014) to aggregate genetic data from across model organisms and advance the translation of evolutionary morphology into a computable format is thus likely to accelerate our understanding of the genetic changes that underlie evolutionarily diverse phenotypes.

## Materials and Methods

### Sources of Data

Evolutionary phenotypes in the Phenoscape Knowledgebase are derived from 52 comparative anatomical studies (Fink SV and Fink WL 1981; Sawada 1982; Gardiner 1984; Schaefer 1987, 1991; Siebert 1987; Mayden 1989; Mo 1991; Cavender and Coburn 1992; Coburn and Cavender 1992; Lundberg 1992; Smith 1992; de Pinna 1993, 1996; Johnson and Patterson 1993; Chen 1994; Friel 1994; Bornbusch 1995; Vari 1995; Vari et al. 1995; Poyato-Ariza 1996; Mooi and Johnson 1997; Bockmann 1998; Buckup 1998; Lucena and Menezes 1998; Malabarba 1998; Shibatta 1998; Soares-Porto 1998; Weitzman and Menezes 1998; Arratia 1999; Di Dario 1999; Grande and Poyato-Ariza 1999; Royero 1999; Toledo-Piza 2000; Wiley et al. 2000; Albert 2001; Sanger and McCune 2002; Britto 2003; Chanet 2003; Chang and Maisey 2003; Fang 2003; Armbruster 2004; Grande et al. 2004; Kailola 2004; Otero 2004; Zaragüeta Bagils 2004; Zanata and Vari 2005; de Pinna et al. 2007; Toledo-Piza 2007; Grande T and Grande L 2008; Sidlauskas and Vari 2008; Vigliotta 2008). These are mostly phylogenetic analyses, with characters and states provided by the original authors in a structured and matrix-based format. Character states were annotated with ontology terms according to the Entity–Quality (EQ) formalism (Washington et al. 2009; Mungall et al. 2010) using Phenex software (Balhoff et al. 2010), in which the entity (E) is typically an anatomical structure that bears a phenotype. Here, entity terms are primarily from the Teleost Anatomy Ontology (TAO; Dahdul, Lundberg, et al. 2010) with some drawn from the GO (Blake and Harris 2008) and others from the Spatial Ontology (Dahdul et al. 2014). Quality (Q) terms, which can be applied to any taxon, were drawn from the PATO (Gkoutos, Green, Mallon, Hancock, et al. 2004; Sprague et al. 2008). EQ phenotypes were associated with taxa using a taxonomy ontology (here the Teleost Taxonomy Ontology; Dahdul, Balhoff, et al. 2010; Midford et al. 2013). Each of these associations, termed “taxon phenotype annotations,” represents part of or a full character state for a taxon. In this way, we created 361,346 taxon phenotype annotations for 2,242 taxa.

Associations of EQ phenotypes with genes, termed “gene phenotype annotations,” were obtained from ZFIN. ZFIN records the phenotypes generated by perturbations of genes, either by mutation or by morpholino knockdown, as reported in the developmental biology literature. These phenotypes are also described using the EQ formalism by using anatomical entities from the Zebrafish Anatomy Ontology, which are treated as species-specific subclasses of the multispecies anatomical entities within the TAO (Dahdul, Balhoff, et al. 2010). At the time of analysis, the Phenoscape Knowledgebase included a total of 11,586 gene phenotype annotations curated in this way, relating to genotypes of 2,966 zebrafish genes. Using this unified ontological framework, the Phenoscape Knowledgebase provides a query interface allowing search of both evolutionary and developmental genetic phenotypes using shared anatomical and phenotypic terms.

### Experimental Validation of In Silico Candidate Gene Predictions

#### Embryo Collection

Three species of catfishes (Teleostei: Siluriformes) were used in this study. *Ictalurus punctatus* (channel catfish) embryos were collected and sent from E.W. Shell Fisheries Research Center (Auburn University, AL) to University of Oregon, Institute of Neuroscience (Eugene, OR). Embryonic suckermouth armored catfish (*Ancistrus* cf. *triradiatus*) and armored bronze corydoras catfish (*C. aeneus*) samples were collected at University of Oregon, Institute of Neuroscience by periodic natural spawning.

Pharyngeal arch developmental timing was established for all three catfishes using samples collected incrementally between 24 and 120 hpf and stained overnight with Alcian Green (Invitrogen, USA). Key time-points in *I. punctatus* pharyngeal arch development were subsequently targeted for *brpf1* in situ hybridization (see below). Endogenous epidermal *eda* and *edar* expression patterns were established for *I. punctatus* using 4-week-old juveniles that originated from the same in vitro fertilization as used for the embryonic time-points. All samples were fixed in 4% paraformaldehyde (PFA) overnight at 4 °C on a rocker, dehydrated using methanol/phosphate-buffered solution (PBS) series and stored at −20 °C in 100% methanol.

#### Cloning

Total RNA was isolated from all collected developmental stages (24–120 hpf) for all three catfishes using TriZol Reagent (Invitrogen) and RNeasy Spin Columns (Qiagen, USA) as per manufacturer’s instructions. Following isolation, total RNA was reverse transcribed into complementary DNA (cDNA) using SuperScriptIII Reverse Transcriptase and oligo dT_20_ universal primers (Invitrogen) following manufacturer’s instructions. Bromodomain containing zinc finger 1 (*brpf1*) and ectodysplasin (*eda*) were isolated from embryonic cDNA using RT-PCR primers (*brpf1*-F: TCGGATATGACATGGACGAG and *brpf1*-R: TAGTGAGGGGCCTCTTGATG; *eda*-F: GTGCTGCAGGATGGGATGTA and *eda*-R: CAAGCTGATTGGCTCACGTA) designed from *I. punctatus* EST sequences ([GH655562] and [FD201975.1]), which are predicted to be orthologs of zebrafish *brpf1* and *eda* ([XP_698063.2] and [XP_001339172.1]), respectively. To isolate ectodysplasin receptor (*edar*), given that no predicted sequence was available in the *I. punctatus* EST database, RT-PCR primers (*edar*-F: AGTGCTGAATACTCGAGCTGT and *edar*-R: TCCAGCCGCTCGATCTGC) were designed using conserved regions of *D. rerio* (NP_001108536.1) and *O. latipes* (NP_001098229.1) nucleotide sequences. Isolated *brpf1*, *eda,* and *edar* RT-PCR products (approximately 750, 450, and 1,300 bp, respectively) were confirmed by sequencing and NCBI BLAST (Basic Local Alignment Search Tool) analyses (Altschul et al. 1990). Verified RT-PCR amplicons were subsequently cloned into PCR4-TOPO vectors according to manufacturer’s instructions (Invitrogen). Clones were sequenced to verify insertion and orientation of target RT-PCR amplicon.

#### *In Situ* Hybridization

Endogenous expression patterns for *D. rerio* and *I. punctatus brpf1* were obtained from whole-mount tissue (72 and 86 hpf embryos, respectively) and *I. punctatus eda* and *edar* were obtained from cryosectioned tissue (4 weeks postfertilization juveniles). Depending on insert orientation (see above), *I. punctatus brpf1*, *eda,* and *edar* digoxigenin-labeled riboprobes were synthesized in vitro using PCR4-TOPO SP6 or T7 promoter following manufacturer’s instructions (Roche, USA). *Danio rerio brpf1* digoxigenin-labeled riborpobes were also synthesized in vitro (Roche) using PCR amplicons (F: ACTGCTACACTGCCTTCCAC; R: TCAGCACCGAGTGTTTCTCC) appended with SP6 or T7 promoter sequence, as detailed elsewhere (Edmunds et al. 2015). Synthesized riboprobes were verified by gel electrophoresis, purified using RNeasy Mini Kit (Qiagen, Inc), quantified by spectrophotometer (Invitrogen, Inc), and diluted in Hyb^+^ buffer (see below) to a final working concentration of 1 ng/μl.

*Brpf1* in situ hybridization was performed on whole-mount tissue using the following protocol modified after Thisse and Thisse (2008): 1) Embryos were collected and fixed in PFA (4%) buffered with 1 × PBS containing 0.1% Tween-20 (PBST) overnight at 4 °C; 2) dehydrated in 100% methanol and transported to Institute of Neuroscience (University of Oregon, Eugene, OR); 3) rehydrated by descending methanol series (75% MeOH/25% PBST, 50% MeOH/50% PBST, 25% MeOH/75% PBST, 100% PBST); 4) dechorionated in PBST; 5) acetone shocked (100% for 10 min at −20 °C); 6) proteinase-K digested (10 μg for 30 min at room temperature); 7) refixed (4% PFA/1 × PBST) at 4 °C overnight; 8) incubated in hybridization solution (Hyb^+^: 50% formamide, 5× sodium sulfanyl citrate [SSC], 50 µg/ml Heparin, 500 µg/ml yeast ribosomal RNA, 0.1% Tween-20, and citric acid to pH 6.0) for 2 h at 68 °C; 9) incubated in pre-warmed Hyb^+^ containing 1 µg *brpf1* antisense probe overnight at 68 °C; 10) washed with Hyb^−^ solution (Hyb^+^ without Heparin, yeast ribosomal RNA or DIG-probe) at 68 °C, as follows: 20 min (75% Hyb^−^ in 25% 2× SSC containing 0.1% Tween-20 [SSCT], 50% Hyb^−^ in 50% 2× SSCT and 25% Hyb^−^ in 75% 2× SSCT), 15 min (100% 2× SSCT in PBST) and 2× 30 min (0.2× SSCT in PBST); 11) cooled to room temperature and washed for 10 min each: 75% 0.2× SSCT in 25% PBST, 50% 0.2× SSCT in 50% PBST, 25% 0.2× SSCT in 75% PBST and 100% PBST; 12) incubated in blocking solution (1× PBS, 1 mg bovine serum albumin [BSA], 2.5 ml inactivated sheep serum, 1 ml goat serum, 0.1% Tween-20) for 1 h at room temperature; 13) incubated in anti-DIG antibody (1:5,000 in blocking solution) at 4 °C overnight; and 14) visualized in color buffer (3.75 μg 4-Nitro blue tetrazolium chloride (NBT), 26.3 μg 5-Bromo-4-chloro-3-indolyl phosphate (BCIP), 0.1 M Tris pH 9.5, 0.05 M MgCl_2_, 0.1 M NaCl, 0.1% Tween-20). Coloration reactions were carried out in the dark at 37 °C until desired degree of labeling.

Similarly, *eda* and *edar* in situ hybridizations were performed on cryosectioned tissues using the following protocol modified after Thisse and Thisse (2008): 1) 4-week-old juveniles were collected and fixed in PFA (4%) buffered with 1× PBST at 4 °C overnight, 2) dehydrated in 100% methanol and transported to Institute of Neuroscience (University of Oregon, Eugene, OR), and 3) rehydrated by descending methanol series (75% MeOH/25% PBST, 50% MeOH/50% PBST, 25% MeOH/75% PBST, 100% PBST). Following rehydration, the body trunk region containing the dorsal fin and torso epidermis was isolated by dissection, embedded in agarose (1%, containing sucrose), and incubated at 4 °C overnight. Cryosections (16 μm) were affixed to poly-lysine-treated slides, immersed in hybridization solution (1× salt solution [1.14 g NaCl, 1.4 g Tris–HCl, 0.13 g Tris Base, 0.78 g NaH_2_PO_4_·2H_2_0, 0.71 g Na_2_PO_4_, 10 ml 0.5 M ethylenediaminetetraacetic acid], 50% formamide, 10% dextran sulfate, 50 mg yeast rRNA, 1× Denhardt’s solution [1% w/v BSA, Ficoll and polyvinylpyrrolidone in water]), and incubated overnight in a humidity chamber (Perspex box with 1× SSC and 50% formamide-wetted Whatman paper). Cryosections were then washed posthybridization for 4× 30 min in wash solution (1× SSC, 50% formamide, 0.1% Tween-20) at 68–70 °C and 3× 30 min in 1× MABT (0.58 g maleic acid, 0.44 g NaCl, 0.1% Tween-20, pH to 7.5 with NaOH), incubated in blocking solution (1× MABT, 2% blocking reagent, 20% heat inactivated sheep serum) for 3 h at room temperature in humidity chamber (Perspex box with 1× PBS-wetted Whatman paper) and then incubated with anti-DIG antibody (1:5,000 in blocking solution) at 4 °C overnight in a humidity chamber (Perspex box with 1× PBS-wetted Whatman paper) before finally being visualized using alkaline phosphate staining buffer (3.75 μg NBT, 26.3 μg BCIP, 0.1 g polyvinyl alcohol, 0.1 M Tris pH 9.5, 0.05 M MgCl_2_, 0.1 M NaCl, 0.1% Tween-20). Coloration reactions were carried out in the dark at 37 °C until desired degree of labeling.

Toluidine Blue Stock Solution (TBSS) was made by dissolving Toluidine Blue powder (1 g; Allied Chemical Co., New York) in 100 ml 70% ethanol. Sodium Chloride Solution (SCS; 1%) was made by dissolving sodium chloride (0.5 g; Allied Chemical Co.) in 50 ml distilled water and adjusting pH to 2.0–2.5 with 4 M HCl. Toluidine Blue Working Solution contained TBSS (5 ml) and SCS (45 ml) at pH 2.3. Dissected arches were stained for 2–3 min, then rinsed (2×) and washed (3×) in distilled water. Counterstained arches were transferred into glycerol by ascending series (25% glycerol/75% PBST, 50% glycerol/50% PBST, 75% glycerol/25% PBST), slide mounted, and imaged (AxioCam MRc-5; Carl Zeiss Inc., Germany).
